# Health Communication during SARS

**DOI:** 10.3201/eid1002.030812

**Published:** 2004-02

**Authors:** Paul M. Arguin, Ava W. Navin, Stefanie F. Steele, Leisa H. Weld, Phyllis E. Kozarsky

**Affiliations:** * Centers for Disease Control and Prevention, Atlanta, Georgia, USA

**Keywords:** severe acute respiratory syndrome, travel, communication, internet

## Abstract

During the severe acute respiratory syndrome (SARS) outbreak, electronic media made it possible to disseminate prevention messages rapidly. The Centers for Disease Control and Prevention’s Travelers’ Health Web site was frequently visited in the first half of 2003; more than 2.6 million visits were made to travel alerts, advisories, and other SARS-related documents.

Experience with the outbreak of severe acute respiratory syndrome (SARS) has reinforced the importance of a multipronged approach to preventing disease transmission. Timely health communication, along with surveillance, quarantine, isolation, and travel restrictions, figured prominently among the tools the Centers for Disease Control and Prevention (CDC) used to help contain the outbreak. During the SARS response, health communication was shown to be an integral element by ensuring that knowledge about prevention measures reached the public, healthcare providers, the media, and other stakeholders.

Disseminating information and educational materials is a key element of CDC’s response to disease outbreaks that affect international travelers. Electronic media greatly expedite the process of dissemination and enable prevention messages to reach an expanded audience. The SARS response may be compared with a situation approximately 10 years before, when an outbreak of plague occurred in India (*1*). In both situations, the challenge was to control a disease outbreak that had potential for rapid international spread and to provide guidance tailored for specific audiences.

## Plague Outbreak, 1994

In late August 1994, CDC received reports from India of an epidemic of plague, the first such outbreak in 24 years. Within 2 months, 5,150 cases of either bubonic or pneumonic plague were reported to the World Health Organization from eight Indian states (*2*). Fifty-six deaths were reported, and >100,000 people fled Surat, a city of approximately 2 million. Neighboring nations closed their borders to travelers and cargo from India, and flights were discontinued.

CDC recognized the need for rapid dissemination of comprehensive educational materials to ameliorate the panic. By the end of September 1994, CDC had produced six documents to distribute to public health officials: an outbreak notice; a plague advisory for travelers to India; a plague alert notice handed to passengers arriving from India, which described the symptoms of plague and urged them to seek medical attention if they developed a febrile illness within 7 days; recommendations for treatment and prophylaxis; guidelines for diagnosis and biosafety; and a review article in the Morbidity and Mortality Weekly Report. These documents were disseminated through an automated fax information service, a voice information service, and a telephone hotline, as well as traditional print media. The fax service reported that 5,589 documents were requested regarding the plague outbreak.

Because of the high volume of air travel from India (approximately 2,000 arriving passengers daily at John F. Kennedy International Airport on flights from India), the departments of health in New York City and New York State supplemented CDC’s surveillance plan by using two approaches to disseminate information to heighten awareness of plague, focusing on emergency department physicians. First, a fact sheet was transmitted by fax or electronic mail to emergency department physicians and infection-control practitioners at 102 hospitals in New York City and to all acute-care hospitals and county health departments in the state. Second, a special plague advisory was distributed to 20,000 physicians in New York City (*3*).

## SARS Response, 2003

**Figure 1:**
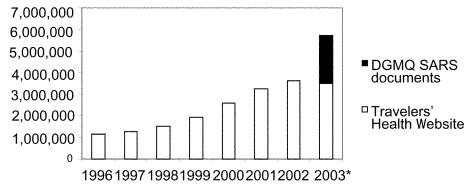
Visits to Centers for Disease Control and Prevention’s (CDC) Travelers’ Health Web site, 1996 through July 2003. * 2003 = Jan–July only, includes documents posted on the CDC SARS Web site as well as the Travelers’ Health website.

The need for educational materials to heighten the awareness of healthcare providers and the public about SARS became obvious early in the outbreak. Because information was rapidly evolving, guidelines needed to be flexible. The “interim” document, one that required constant updating, became the norm. The Internet became a primary tool for communication, as it has been for CDC travelers’ health information. In fact, before the SARS outbreak, the travelers’ health Web site (located within the CDC Web site; available from: URL: www.cdc.gov\travel) had become the most frequently visited CDC Web site other than the home pages, with more than 3.6 million visits recorded in 2002 (Figure 1). Visits to the Web site increased dramatically in 2003. As of July, >4 million visits had been recorded to the travelers’ health Web site; more than 1 million of these visits resulted from accessing SARS-related content (travel alerts and advisories). Although the target audience for this Web site is in the United States, approximately one third of the visits were from other countries. In May, the city from which the most visits originated was Taipei, Taiwan, with more visits than any city in the United States. The SARS-related documents were not posted in multiple areas on the Web site but could be accessed by navigating through the Web site using different routes. Data from Web-tracking software showed that approximately 83% of visitors came from a commercial or .net domain, 10% from educational domains, 3% from .org domains, 2% from government domains, and 1.5% from military domains.

As part of the SARS response, CDC’s Division of Global Migration and Quarantine (DGMQ) developed travel-related information and recommendations, as well as industry-specific guidelines. Web sites that referred to these pages with a substantial number of visits included those from 1) organizations serving constituent groups such as families adopting children from Asia and expatriates overseas, 2) organizations with major meetings or conferences in areas with SARS, and 3) major news organizations. Overall, during the outbreak, DGMQ generated >125 documents, including updates and translations into seven languages, which were posted on the SARS pages of the CDC Web site. This material was written for multiple audiences, from highly technical to low literacy, and was disseminated through multiple platforms, from traditional print (e.g., >2,700,000 yellow Health Alert Notices were handed out by Quarantine Officers to passengers disembarking from 11,840 flights from areas with SARS) to electronic (postings on Web sites and CDC’s Secure Data Network).

As the outbreak matured and additional stakeholders were identified, interim guidelines were tailored to the specific concerns of healthcare providers, industry, and the traveling public (Table). Fact sheets explaining the legal authority for isolation and quarantine were written and posted. More than 1.5 million visits were made to DGMQ documents on CDC’s SARS Web site, in addition to the 4 million visits to the Travelers’ Health Web site.

**Table:** SARS-related documents generated by the Division of Global Migration and Quarantine, Centers for Disease Control and Prevention, March–July 2003^a^

Category	Document	Mo. of initial version	URL
Travelers/Public	Interim travel advisories and alerts	March	http://www.cdc.gov/travel/
	Health Alert Notice (in 7 languages)	March	http://www.cdc.gov/ncidod/sars/travel_alert.htm
	**Interim definitions and criteria: travel alerts vs. travel advisories**	May	http://www.cdc.gov/ncidod/sars/travel_alertadvisory.htm
	Interim guidelines about SARS for persons traveling to areas with SARS	April	http://www.cdc.gov/ncidod/sars/travel_advice.htm
Legal and Quarantine	**Fact sheet**: i**solation and quarantine**	April	http://www.cdc.gov/ncidod/sars/isolationquarantine.htm
	**The SARS investigation: the role of CDC’s division of global migration and quarantine**	March	http://www.cdc.gov/ncidod/sars/roleofdq.htm
	**Questions & answers: travel and quarantine**	April	http://www.cdc.gov/ncidod/sars/qa/travel.htm
	**Fact sheet on legal authorities for isolation/quarantine**	April	http://www.cdc.gov/ncidod/sars/factsheetlegal.htm
Industry Specific Guidelines	Interim guidelines about severe acute respiratory syndrome (SARS) for airline flight crew members	March	http://www.cdc.gov/ncidod/sars/flight_crew_guidelines.htm
	Interim guidelines for cleaning of commercial passenger aircraft following a flight with a passenger with suspected SARS	March	http://www.cdc.gov/ncidod/sars/aircraftcleanup.htm
	Interim guidelines for personnel interacting with passengers arriving from areas with SARS	March	http://www.cdc.gov/ncidod/sars/tsa-bcbp-guidelines.htm
	Interim guidelines about SARS for cruise ship passengers and crew members	April	http://www.cdc.gov/ncidod/sars/cruiseship.htm
	Interim guidelines for personnel boarding maritime vessels from areas with SARS	May	http://www.cdc.gov/ncidod/sars/maritime.htm
	Interim guidelines about SARS for workers handling cargo or other packages	May	http://www.cdc.gov/ncidod/sars/cargoworkers.htm
	**Interim guidelines and recommendations: prevention, identification and management of suspect & probable cases of severe acute respiratory syndrome on cruise ships**	May	http://www.cdc.gov/ncidod/sars/cruiseshipguidelines.htm
Other	**Interim guidance for institutions or organizations hosting persons arriving in the United States from areas with severe acute respiratory syndrome (SARS)**	May	http://www.cdc.gov/ncidod/sars/hostingarrivals.htm
	**Interim guidelines for businesses and other organizations with employees returning to the United States from areas with** **SARS**	May	http://www.cdc.gov/ncidod/sars/business_guidelines.htm
	**Interim guidelines about SARS for international adoptees and their families**	March	http://www.cdc.gov/ncidod/sars/adoption.htm
	**Guidance about SARS for Americans living abroad**	March	http://www.cdc.gov/ncidod/sars/warden_notice.htm
	**Interim guidance: air medical transport for severe acute respiratory syndrome (SARS) patients**	**March**	**http://www.cdc.gov/ncidod/sars/airtransport-sarspatients.htm**

^a^Documents were updated and revised multiple times

**Figure 2:**
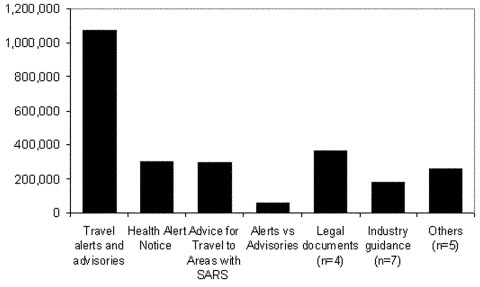
Visits to SARS-related documents posted by Division of Global Migration and Quarantine on Centers for Disease Control and Prevention Web site, January–July 2003.

The travel alerts and advisories received the most visits (Figure 2). Historically, CDC has never advised against travel to any region, even during the plague epidemic in India. However, because of the rapid spread of SARS, its short incubation period, and the potential severity of illness, the need was recognized to codify different levels of concern about potential transmission to travelers. Thus, the travel alert and advisory system was developed.^1^

A travel alert is a notification by CDC that an outbreak of a disease is occurring in a geographic area. Its purpose is to provide information to travelers and resident expatriates about the status of an outbreak, how to reduce their risk for infection, and what to do if they become ill. The risk for individual travelers is thought to be definable and limited. In contrast, a travel advisory recommends against nonessential travel to an area because the risk to travelers is considered to be high as a result of ongoing transmission or inadequate containment. The travel advisory not only provides information about the status of an outbreak, but also is intended to reduce risk for exposure by decreasing the volume of traffic to the affected area.

These designations were used for the first time during the SARS outbreak, and thus criteria for their introduction, downgrading, and removal were required. Institution of either an alert or advisory was dependent on the magnitude and scope of the outbreak, the containment measures being used, the quality of surveillance in the affected area, and the quality and accessibility of medical care, all of which are based on reports from the involved countries. Once instituted, downgrading an advisory to an alert required adequate surveillance and no evidence of ongoing transmission for at least two incubation periods after the date of onset of symptoms in the last case (for SARS, 20 days). Removing an alert was dependent on the above criteria, as well as lack of evidence of new cases for three incubation periods (for SARS, 30 days) and no exportation of cases, as determined by an assessment of the information reported from the countries involved.^2^

During the outbreak, the relationship between DGMQ and the airline industry through the Airline Transport Association (ATA) and the airline medical directors was strengthened. As international spread of SARS through airline travel became a possibility, ATA was not only eager to provide information necessary for tracking passengers, but also served as a sounding board for specific guidelines for the traveler, flight crew, cargo handlers, and cleaning crew, and for the management of ill passengers. Other stakeholders included the cruise ship industry and U.S. citizens living overseas.

## Conclusions

A comparison of the efforts in mass communication during the Indian plague outbreak that occurred in 1994 with those during SARS is illustrative of the changes that have resulted from the large increase in numbers of travelers, the decreased time in transiting the globe, and the massive demand for instant information (*4*). Electronic communications media enabled information to reach much wider audiences than had been possible through means such as traditional print media and fax services and allowed distribution of guidelines directed at specific target audiences. During the 1994 plague outbreak, thousands of documents were distributed by traditional means; during the SARS response, which lasted approximately the same time, millions of documents were disseminated through the CDC Web site.

## Footnotes

1 In the 1994 plague documents, the term “advisory” did not have the same connotation.

2 These criteria differed from those used by the World Health Organization.
